# The first complete mitochondrial genome of the Grey-bellied Green Ratsnake *Gonyosoma frenatum* (Gray, 1853) and its phylogenetic placement

**DOI:** 10.1080/23802359.2021.1959436

**Published:** 2021-11-10

**Authors:** Le Wang, Bin Cai, Yan-Bin Huang, Hai-Ping Shangguan, Yan-Qing Wu

**Affiliations:** a Ministry of Ecology and Environment, Nanjing Institute of Environmental Sciences, Nanjing, China; b Scientific Monitoring Center, Administration Bureau of National Park of Wuyi Mountain, Wuyishan, China; c Administration Bureau of Fujian Junzifeng National Nature Reserve, Mingxi, China

**Keywords:** Colubridae, *Gonyosoma frenatum*, mitochondrial genome, phylogenetic relationship, next-generation sequencing

## Abstract

We sequenced the complete 17,209 bp mitochondrial genome (mitogenome) of *Gonyosoma frenatum* (Squamata: Colubridae) using next-generation sequencing. It consists of 13 PCGs, two ribosomal RNA genes, 22 transfer RNA genes, one non-coding region of an L-strand replication origin, and two control regions. The overall nucleotide composition was 34.7% of A, 24.8% of T, 12.3% of G, and 28.1% of C. The result of the phylogenetic analysis showed that *G. frenatum*, a member of Colubridae, is sister to other New World and Old World ratsnakes. The new data could help better understand the phylogenetic status of the genus *Gonyosoma* and the evolutionary history of Colubridae species.

*Gonyosoma frenatum* (Gray, 1853) is an arboreal snake with a green dorsal surface and a white belly, and its head has a lateral black stripe from the nostril across the eye to the neck (Zhao 2006). This species has a wide distribution in China, India and Vietnam (Pham et al. 2014). However, the classification of this genus is still unclear mostly because of inadequate representation in molecular phylogenies (Pyron et al. 2013; Chen et al. 2014). No complete mitochondrial genome (mitogenome) of any *Gonyosoma* species has been published so far. Here, we obtained and present the complete mitogenome of *G. frenatum* via next-generation sequencing.

The specimen of *G. frenatum* was collected from Ziyun village (26.363322°N, 117.474934°E) in Mingxi county, Fujian province, China. The specimen (specimen number: LSU20200812JZF01) was deposited in the Museum of Laboratory of Amphibian Diversity Investigation of Lishui University. Total genomic DNA was extracted from muscle tissue using the EasyPure Genomic DNA Kit (TransGen Biotech Co, Beijing, China). The raw sequence data (14.55 G) was deposited in NCBI’s Sequence Read Archive (SRA; accession: SRR13309601), and the sequencing of the genomic library (14.52 G) was carried out on an Illumina NovaSeq6000 platform (Novogene Bioinformatics Technology Co. Ltd., Tianjin, China) with a paired-end 150 bp approach. The mitogenome was assembled with NOVO Plasty 3.7 (Dierckxsens et al. 2017), and we using the known mitochondrial genes of *G. frenatum* (*COI* MK064641, *Cytb* DQ902110, *ND1* DQ902158, *ND2* DQ902217, *ND4* DQ902290) as reference genome. Finally, the mitogenome annotated using the MITOS Web Server (Matthias et al. 2013).

In this study, the total length of the *G. frenatum* mitogenome is 17,209 bp. We uploaded this circular mitochondrial genome in NCBI (MW413812). It includes 13 protein-coding genes (PCGs), two ribosomal RNA (rRNA) genes, 22 transfer RNA (tRNA) genes, one non-coding region of an L-strand replication origin and two control regions (D-loop1 and D-loop2). D-loop1 was located between two tRNAs (trnI and trnL2) with a length of 1060 bp. The overall nucleotide composition was 34.7% of A, 24.8% of T, 12.3% of G and 28.1% of C. There were 12 PCGs, 14 tRNA genes and two rRNA genes encoded on the H-strand. The ND6 and eight other tRNA genes were encoded on the L-strand. Among the 13 PCGs, ND5 was the longest (1773 bp) and ATP8 was the shortest (159 bp). Codon usage analysis showed that 12 PCGs started with a typical ATN codon (one with ATT, two with ATA, and nine with ATG), whereas, the COX1 gene appeared to start with GTG. Seven PCGs ended with complete stop codons (four with TAA, one with TAG, one with AGA, and one with AGG). Six PCGs (COX2-3, ND1-3, and CYTB) ended with incomplete stop codons T– (TA-), which could form TAA by post-transcription polyadenylation (Anderson et al. 1981).

We constructed phylogenetic relationships based on the 13 PCGs (11,274 bp) for 15 Colubrinae species, and used *Stichophanes ningshaanensis* (Squamata: Colubridae) as the outgroup. The optimal substitution model (GTR + I + G) was selected via jModelTest (Darriba et al. 2012). Bayesian inference (BI) was utilized in MrBayes v3.2.7 with four parallel runs of Markov Chain Monte Carlo (MCMC) analyzed for 1,000,000 generations. This analysis sampled every 1000 generations and discarded 1000 trees as burn-in, and the analyses were considered to have converged when the average standard deviation of split frequencies below 0.01. The phylogenetic tree showed that *G. frenatum* is positioned as a sister clade to other New World and Old World ratsnakes (Figure 1). This first complete mitogenome sequence of *Gonyosoma* could help better understand the evolutionary history of the family Colubridae.

**Figure 1.**
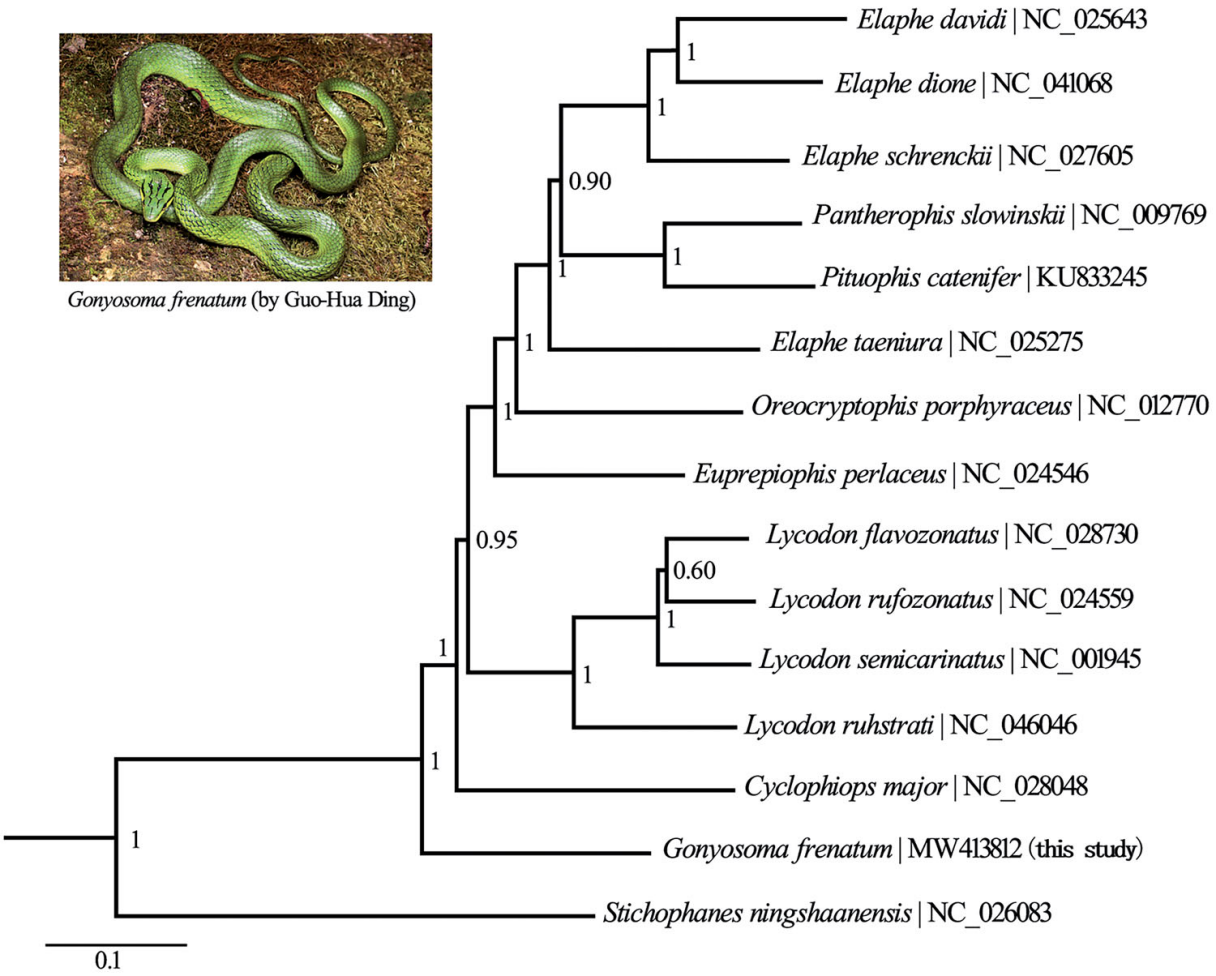
Phylogenetic tree obtained from the BI analysis, based on 13 concatenated mitochondrial PCGs. Numbers on the nodes are posterior probability values.

## Data Availability

The mitogenome data supporting this study are openly available in GenBank at [https://www.ncbi.nlm.nih.gov/nuccore/MW413812]. Reference number [Accession number: MW413812]. BioSample and SRA accession numbers are [https://www.ncbi.nlm.nih.gov/biosample/SAMN17167288], [https://www.ncbi.nlm.nih.gov/sra/SRR13309601], respectively.
